# A Phenomenological and Clinical Description of Pandemic Grief: How to Adapt Bereavement Services?

**DOI:** 10.1089/pmr.2022.0060

**Published:** 2023-03-13

**Authors:** Melanie Vachon, Deborah Ummel, Alexandra Guité-Verret, Emilie Lessard, Dominique Girard

**Affiliations:** ^1^ Department of Psychology, Université du Québec à Montréal, Montréal, Québec, Canada.; ^2^ Center for Research and Intervention on Suicide, Ethical Issues and End-of-Life Practices (CRISE), Montreal, Québec, Canada.; ^3^ Réseau Québécois de Recherche en Soins Palliatifs et de fin de vie (RQSPAL), Québec, Canada.; ^4^ Department of Psychoeducation, Université de Sherbrooke, Longueuil, Québec, Canada.; ^5^ University of Montreal Hospital Research Center, Montreal, Québec, Canada.; ^6^ Department of Anesthesiology, Pain and Palliative Medicine, Radboud University Medical Center, Nijmegen, the Netherlands.

**Keywords:** bereavement services, COVID-19, interpretative phenomenological analysis, pandemic grief

## Abstract

**Background:**

Some studies suggest that individuals having lost a loved one during the COVID-19 pandemic report higher levels of grief reactions than people bereaved from natural causes. Little is known about the lived and subjective experience of individuals who lost a loved one under confinement measures.

**Aim:**

This research aims to provide a phenomenological description of pandemic grief (PG) that can be useful in clinical settings and bereavement services.

**Methods:**

Seventy-six qualitative phenomenological interviews have been conducted with 37 individuals who have lost a loved one during the first wave of the pandemic. Interpretative phenomenological analysis was performed following Tracy's criteria for rigorous qualitative research.

**Results:**

The experience of PG comprises clinical manifestations and can be described as “a type of grief occurring in the context of a pandemic, where applicable public health measures have precedence over end of life and caregiving practices as well as funeral rituals, overshadowing the needs, values, and wishes of the dying individuals and those who grieve them.”

**Discussion/Conclusion:**

This study is the first to provide a phenomenological and experiential understanding of PG. Our phenomenological description can be helpful in clinical settings such as bereavement services within palliative care teams.

## Introduction

Confinement measures put into place to slow down the COVID-19 pandemic have tragically disrupted the conditions of individuals at the end of their lives and, therefore, the bereavement process of their family caregivers.^1^ Indeed, recently published studies suggest that individuals having lost a loved one during the pandemic report higher levels of grief reactions than people bereaved from natural causes.^2^

Palliative care (PC) aims at caring for dying patients and their loved ones from diagnosis to bereavement. Psychosocial professionals working in multidisciplinary PC teams providing bereavement services should be prepared to intervene with individuals presenting particular symptoms given the death circumstances of their loved one (e.g., feelings of guilt and regret). Even if scales measuring the severity of “pandemic grief” (PG) have been developed,^3^ little is known about the lived experience of individuals who lost a loved one under confinement measures. Therefore, this research aims to provide a phenomenological description of PG that can be useful in clinical settings and bereavement services.

## Methods

### Participants and study design

Participants were grieving individuals under confinement measures recruited in Quebec, Canada. They were recruited using Facebook and the snowball technique. Most participants spontaneously joined the project by word of mouth.^†^ Inclusion criteria consisted of being at least 18 years old, reading, understanding, and speaking French, and having lost a loved one during the first wave of the pandemic (March–December 2020). Concerning ethics, we conducted the research according to the institutional review board approval from Université du Québec à Montréal (UQAM).

Participants' characteristics are displayed in Table 1. The final sample comprised 37 grieving individuals who lost a loved one from COVID-19 or severe illness and received PC at the end of their life during the first wave of the pandemic (March–December 2020). Some participants lost more than one family member during this period. More than half of our participants wished to participate in more than one interview, as they had a lot to share with us (the number of interviews per participant varied between one and five).

**Table 1.** Participant Characteristics

	***N***	Mean	Range
Age		58	21–78
Length of interviews (minutes)		61	44–94
Number of interviews			
1	20		
2	11		
3	3		
4	1		
5	2		
Time since death (weeks)		17	15–45
Gender			
Women	33		
Men	4		
Relationship with the deceased			
Mother	15		
Father	11		
Spouse	5		
Grand parent	4		
Sibling	2		
Place of death			
CHSLD	9		
Hospital	24		
At home	4		
Cause of death			
Following a COVID-19 diagnostic	26		
Severe illness	11		
Place of origin			
Canada	32		
America	1		
Europe	1		
Africa	3		
Religious affiliation			
Christian (Catholic or Protestant)	16		
Judaism	1		
Not specified	20		

*N* total = 37.

CHSLD, long-term care facility.

A total of 76 in-depth individual interviews were conducted through videoconference by clinical psychologists with expertise in both PC and phenomenological interviewing. Questions were open ended and focused on the whole grief trajectory. We invited the participants to describe their personal experiences from the question: “Can you tell me what happened to you from the beginning of the pandemic until today?” Death circumstances and care received at the end of life were also explored during the interviews. The interview was a co-construction in which responses were probed when needed with reflections and follow-up questions. Reflective notes were taken after each interview and discussed between interviewers and authors.

### Data analysis

We verified, transcribed, and analyzed^4^ all the interview data following the main principles of interpretative phenomenological analysis (IPA).^5^ IPA is a flexible and iterative process rather than a rigid and linear method.^5,6^ The seven suggested steps were followed: (1) reading and rereading the first case, (2) initial noting, (3) developing emergent themes, (4) searching for connections across emerging themes, (5) moving to the next case, (6) repeating steps 1 to 4 for each case, and (7) looking for patterns across cases.

Congruently with the “double hermeneutic” of the IPA theoretical foundations, we interpreted how each participant expressed and interpreted the phenomenon of grieving in the context of the COVID-19 pandemic. In line with this idiographic approach, we considered each case individually before analyzing the convergence and divergence between cases. As the hermeneutic circle speaks to nonlinear analysis, our understanding remains open to new insights. It was up to researchers to decide when the interpretation was satisfactory.^7^ We followed Tracy's^8^ criteria for rigor in qualitative research.

## Results

The final analysis allowed us to describe the essence of the experience of PC as “a type of grief occurring in the context of a pandemic, where applicable public health measures have precedence over end of life and caregiving practices as well as funeral rituals, overshadowing the needs, values, and wishes of the dying individuals and those who grieve them.” The individual's PG experience may include one or several of the following seven characteristics (Table 2):

**Table 2.** Example of Citations

Theme	Quote
Feeling remorse, regrets, or guilt	*I haven't been able to see her. Sometimes, I'm thinking, maybe if I took advantage of my contacts, if I had tried to push everything [the rules], if Mommy had seen me… Maybe she wouldn't have deteriorated… P2*
Having traumatic memories or pictures	*I see his leaner face again. I couldn't recognize him. It's all coming back to me, those images… P20*
Feeling anger, injustice, frustration, and/or indignation	*I am at peace with the fact that he is deceased, but I can't make peace with the way this happened. Those older people have been left to themselves. P22*
Experiencing difficulties in getting back to the “normal” flow of life	*Bereavement is one thing. And there is the lockdown on top of it. We cannot go through our grief as we would normally have. P3*
Experiencing solitude in suffering, specific to PG	*People do not understand. I said quickly that I was going through a difficult time, that my mother died during this COVID period… They asked me: ‘how old was she?’. ‘88 years old’, I answered. They told me: ‘Oh, you know, they [older people] have to go at some point’. I found her comment… We quickly changed the subject. P6*
Feeling shocked	*It is an enormous shock. Indeed, he wasn't doing so bad… but he got COVID and we didn't see him again. He died soon after, suddenly. We couldn't even realize that he was not there anymore. P24*
Being in a prolonged liminal state	*There's something absolutely unresolved until today. It's really unfinished. I have days where I know she died and days where it's fuzzier, still up to now. It's a non-resolution, and we are still waiting… P18*

- **Feeling remorse, regrets, or guilt** regarding the circumstances of death (inability to accompany the dying person, limited contact with others, etc.) and/or the funeral rituals that did not respect the deceased's last wishes.
- **Having traumatic memories or pictures** associated with the death or the steep decline of the loved one (leaner face, bearing witness to the mishandling of the corpse, etc.)
- **Feeling anger, injustice, frustration, and/or indignation** toward authorities responsible for public health measures or the managers or health care providers where the loved one died.
- **Experiencing difficulties in getting back to the “normal” flow of life**: the saturated media context of the pandemic forced individuals to relive their loss experience constantly. Furthermore, public health measures deprived individuals of prepandemic “normality.” Personal and professional support sources and possibilities to encounter positive experiences in everyday life were more limited.
- **Experiencing solitude in suffering, specific to PG**. Feeling that the grief was banalized or its importance was diluted given the number of simultaneous deaths. This feeling of grief and nonrecognition of loss could have been accentuated by the lack or postponement of commemorations and/or by the restrictions imposed on such commemorations (limited physical contact, limited guest list, etc.)
- **Feeling shocked** and experiencing a lack of understanding at the time of death and the following weeks. A transitory impression of unreality sometimes accompanied these feelings.
- **Being in a prolonged liminal state** between two worlds (pre- and post-COVID). This feeling of being caught in the middle was marked by the uncertainty regarding the end of life and dying trajectory (not having witnessed the other's decline, not having seen the deceased body, etc.). The immateriality of the loss also marked this liminal state, given the lack of significant funeral rituals allowing for the grieving process to emerge.

## Discussion

The COVID-19 pandemic plunged millions of individuals into grief. Recent studies suggested that bereaved individuals who lost a loved one during the pandemic may present with severe and specific grief reactions.^2,3^ Psychosocial professionals in PC bereavement services must better understand the experience of PG to adapt their interventions. This research provides a complete, experiential, and clinically helpful description of the essence of PG and its clinical manifestations.

Typical interventions to support grief should be adapted for individuals who lost a loved one during the pandemic in a way that is adapted to the specific manifestations they are presenting.^9–12^ For example, putting into words the grief experience could allow for the recreation of a coherent narrative of this experience and alleviate the feeling of shock, even traumatic pictures. Narration and therapy based on meaning-making^9^ could also allow bereaved individuals to find a new meaning to their experience, revisit painful emotions including but not restricted to regrets, remorse, guilt, and anger, and cocreate new meanings out of their experience.^10,11^

This study was part of a larger research-intervention project. In the context of this larger project, we experimented with how art could represent a powerful alternative in supporting bereaved individuals from the pandemic in the grieving process. Indeed, artistic creation can translate an emotional reality in pictures and symbols, allowing and supporting the operation of the integration of the loss.^13^ Art is also a cultural mediator through which individuals can connect with their grieving experience. As such, it becomes possible to break isolation and solitude and to encourage the social recognition of PG.^10–12^ Our team is currently working on publications focusing on art-based interventions in the context of PG.

## Conclusion

This study is the first to provide a phenomenological and experiential understanding of PG. Our findings are limited to our particular sample, which is self-selected and very homogenous in terms of sociodemographic characteristics. Despite these limitations, our definition can be helpful in clinical settings such as bereavement services within PC teams. Further studies may address some clinical particularities in working with bereaved individuals who lost a loved one in PC during the pandemic.

## Abbreviations Used

IPA: interpretative phenomenological analysis
PC: palliative care
PG: pandemic grief

## Notes

^†^
The province of Quebec declared a state of health emergency on March 13, 2020. Confinement measures included the complete prohibition of outside caregivers visiting hospital centers and different types of residential aged care facilities maintained until May 2020. Public health measures have also impacted funeral ceremonies and other commemorative rituals, for instance, the prohibition of any indoor or outdoor gathering. Funerals rituals were first strictly reserved for a “familial bubble.” Later, these measures became more flexible, with several persisting constraints such as a limited number of people (e.g., only 25 people in a red zone), the requirement to wear a procedure mask, and to maintain the whole time a 2 m distance between individuals who were not living at the same address.
